# Autophagy is induced in human keratinocytes during human papillomavirus 11 pseudovirion entry

**DOI:** 10.18632/aging.104046

**Published:** 2020-11-16

**Authors:** Rui Han, Chunting Hua, Siyuan Sun, Boya Zhang, Yinjing Song, Stijn van der Veen, Hao Cheng

**Affiliations:** 1 Department of Dermatology and Venereology, Sir Run Run Shaw Hospital, School of Medicine, Zhejiang University, Hangzhou, China; 2 Department of Microbiology and Parasitology, Collaborative Innovation Center for Diagnosis and Treatment of Infectious Diseases, School of Medicine, Zhejiang University, Hangzhou, China

**Keywords:** human papillomavirus 11, entry, autophagy, keratinocyte, pseudovirion

## Abstract

Human papillomavirus type 11 (HPV11) is one of the main causes of condyloma acuminatum, a widespread sexually transmitted disease. During infection of its primary target cell, keratinocytes, it is likely to encounter the autophagy pathway, which is an intracellular maintenance process that is also able to target invading pathogens. It is currently unknown whether HPV11 is targeted by autophagy or whether it is able to escape autophagy-mediated killing. Here, we investigated the autophagy response during HPV11 pseudovirion (PsV) entry in human keratinocytes. Transmission electron microscopy showed that intracellular PsVs were sequestered in lumen of double-membrane autophagosomes that subsequently appeared to fuse with lysosomes, while confocal microscopy showed induction LC3 puncta, the hallmark of induced autophagy activity. Furthermore, quantitative infection assays showed that high autophagy activity resulted in reduced HPV11 PsV infectivity. Therefore, the autophagy pathway seemed to actively target invading HPV11 PsVs for destruction in the autolysosome. Western analysis on the phosphorylation state of autophagy regulators and upstream pathways indicated that autophagy was activated through interplay between Erk and Akt signaling. In conclusion, autophagy functions as a cellular protection mechanism against intracellular HPV11 and therefore therapies that stimulate autophagy may prevent recurrent condyloma acuminatum by helping eliminate latent HPV11 infections.

## INTRODUCTION

Human papillomaviruses (HPVs) are small non-enveloped double-stranded DNA viruses that strictly infect the mucosal and cutaneous epithelium [1, 2]. Over 200 HPV genotypes have been identified thus far. Based on their pathogenicity, HPVs can be classified into low-risk and high-risk types [3]. The low-risk HPVs, particularly HPV type 6 and HPV type 11, are the main causes of condyloma acuminatum (genital warts), which has become one of the most widespread sexually transmitted diseases [4, 5]. Persistent infection and recurrence are the major difficulties in managing HPV infections, since host cellular immunity is insufficiently induced for effective clearance of the HPV [6, 7].

HPV genomic DNA is encased by a ~55 nm icosahedral capsid, which is comprised of two capsid proteins, protein L1 (major) and L2 (minor), encoded by the late genes [8]. Like many viral pathogens, HPV initiates entry by binding to target cells through interactions between L1 and heparan sulfate proteoglycans (HSPGs), triggering conformational changes in both L1 and L2 capsid proteins [9–12]. Subsequent steps in cellular entry remain poorly understood.

Autophagy has essential functions in cell homeostasis, as autophagosomes engulf bulk cytoplasm, including damaged organelles, and shuttle them to lysosomes for degradation and recycling of nutrients [13]. Autophagy is known to play roles in survival against starvation, quality control of intracellular proteins and organelles, anti-aging, suppression of tumor formation, antigen presentation and elimination of intracellular microbes [14]. As a part of the host defense system, autophagy likely interacts with intracellular viruses throughout the course of infection. Autophagy, however, can play both anti- and pro-viral roles, depending on the specific virus, the cell type, and the cellular environment [15, 16]. Autophagy proteins function in targeting viral components or virions for lysosomal degradation in a process termed xenophagy, which is an important component of the host response against viral infections [17]. However, some viruses appear to utilize or hijack components of the autophagic machinery to improve viral replication [18]. For HPVs, it has been reported that high-risk HPV type 16 entry modulates an autophagy response, including regulation of marker proteins for autophagy in keratinocytes [19, 20]. However, the autophagy response to cellular entry by low-risk HPV types is currently unknown.

In this study, we show that autophagy is activated upon entry of HPV type 11 pseudovirion (PsV) particles into human keratinocytes. Moreover, in primary keratinocytes (NHEKs), which display low levels of basal autophagy, HPV11 PsV entry induces lower autophagic activity and weaker Erk (extracellular signal-regulated kinase)/mTOR inhibition compared with immortalized keratinocytes (HaCaT cells) that display higher basal autophagy levels. Furthermore, HPV11 PsV infectivity is much higher in NHEK cells compared with HaCaT cells and significantly inhibited by rapamycin. Our results suggest that host autophagy is induced by HPV11 PsV entry and functions as a host defense mechanism to protect against HPV11 infections.

## RESULTS

### Subcellular localization of HPV11 PsV particles

**Figure 1:**
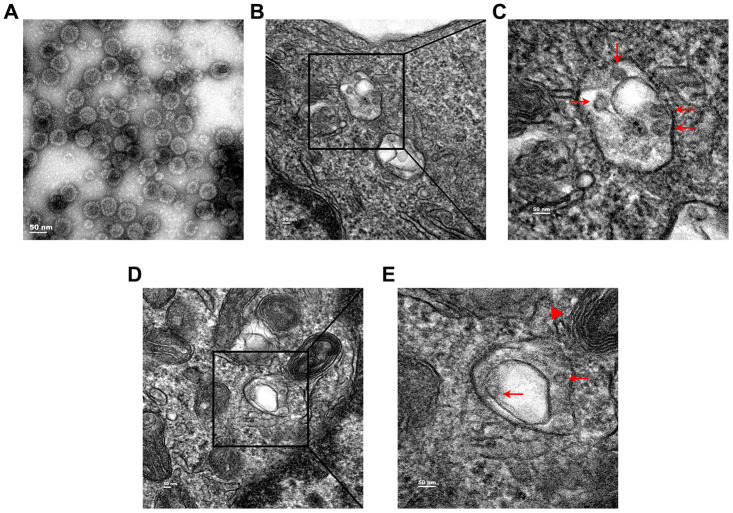
**Transmission electron microcopy (TEM) analysis of subcellular localization of HPV11 PsV particles in NHEK cells.** (**A**) TEM images of purified HPV11 PsV particles. (**B**, **C**) HPV11 PsV particles appear in the lumen of double-membrane vesicle. (**D**, **E**) Engagement of HPV11 PsV particles-containing autophagosome by a lysosome. Arrows indicate HPV11 PsV particles and the triangle indicates a lysosome.

To confirm the quality of HPV11 PsV particles, virion morphology was visualized by transmission electron microscopy (TEM), which showed homogeneous-sized particles of approximately 55 nm in diameter (Figure 1A). As the natural host target-cell for HPV infections, primary human keratinocytes have been accepted as one of the best cell culture models to study HPV infection mechanism. Thus, normal human epidermal keratinocytes (NHEKs) were incubated with HPV11 PsVs for 4h at 37 °C and intracellular localization PsV was visualized by TEM. Intracellular electrondense PsV particles of approximately 55 nm were commonly observed in the lumen of double-membrane vesicles (Figure 1B and 1C) that were frequently engaged by lysosomes (Figure 1D and 1E). Therefore, these results suggest that intracellular HPV11 PsVs are targeted by autophagy.

### LC3 puncta are increased upon HPV11 PsV infection

**Figure 2:**
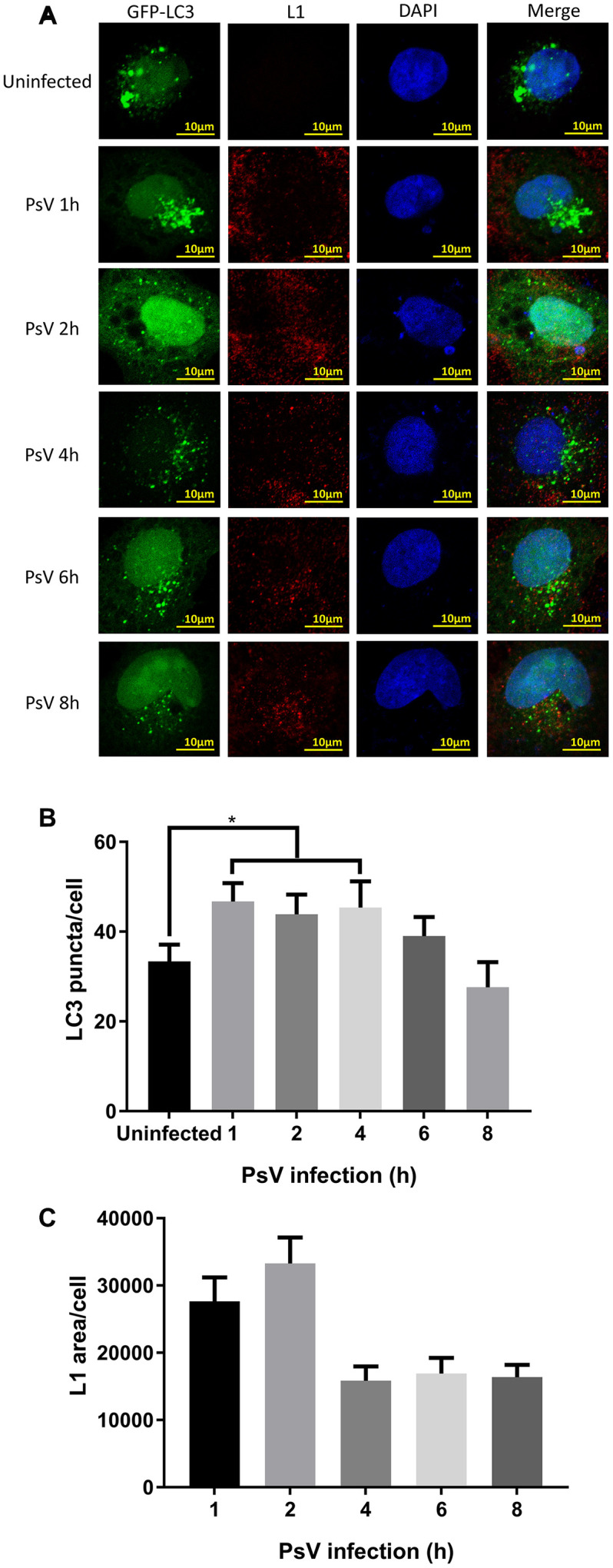
**LC3 puncta are increased after HPV11 PsV infection of NHEK cells.** (**A**) Confocal microscopy analysis of GFP-LC3 puncta and L1-stained HPV11 PsVs. The NHEK nucleus was stained with 4’,6-diamidino-2-phenylindole (DAPI). (**B**) Quantification of GFP-LC3 puncta in panel (**A**). (**C**) Quantification of the total L1 staining area in panel (**A**). The graphs represent the mean and standard deviation of three biological independent experiments, and at least 100 cells were analyzed for each repeat. Significant differences were identified by Student’s t test. *, P < 0.05.

LC3 is one of the most frequently used autophagy activity marker proteins and its clustering, visualized by the formation of puncta, is indicative of phagophore and autophagosome formation. Therefore, NHEKs were transfected with a plasmid expressing GFP-LC3 and subsequently used in HPV11 PsV infection experiments. LC3 puncta were visualized after 1, 2, 4, 6, and 8 hours of infection, while PsVs were visualized by immunostaining of L1 (Figure 2A). After infection, the number of LC3 puncta was significantly elevated compared with uninfected cells and only after 4 hours of infection the numbers gradually reduced (Figure 2B). Similarly, quantification of L1 fluorescent areas showed the maximum PsV levels were reached after 2 hours of infection, while after 4 hours the PsV levels appeared to plateau at a lower basal level without showing further reductions (Figure 2C). Importantly, co-localization between LC3 puncta and PsVs/L1 appeared to be limited. However, this might be explained by the acid-sensitivity of GFP, which loses its fluorescence in the acid environment of autolysosomes. Overall, these data indicate that PsV entry into NHEKs results in activation of autophagy.

### Recycling of LC3-II and p62 is promoted by HPV11 PsV entry

**Figure 3:**
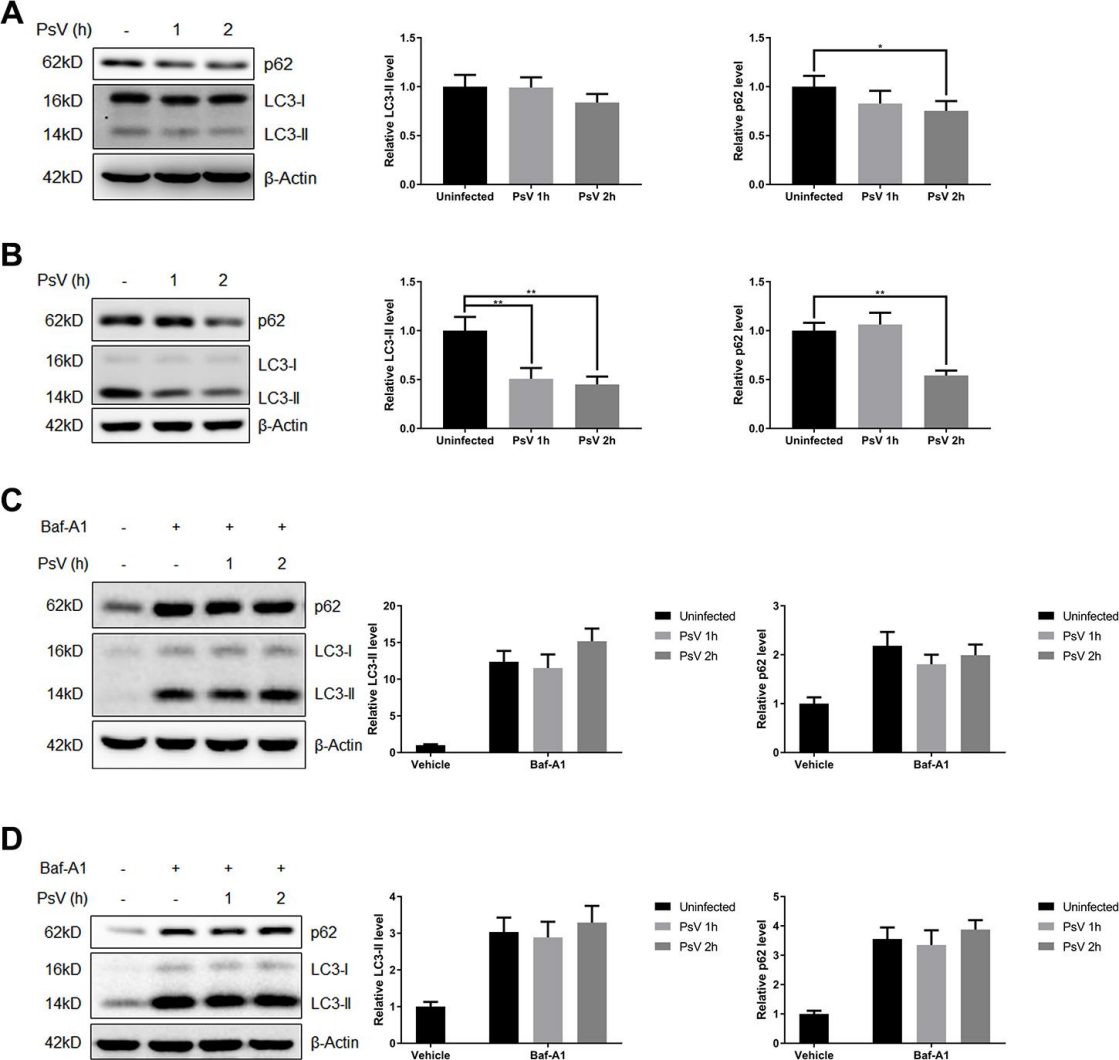
**Autophagy flux is promoted by HPV11 PsVs upon entry of keratinocytes.** (**A**, **B**) Western analysis and quantification of the autophagy marker proteins p62 and LC3 in NHEKs (**A**) or HaCaT cells (**B**) during HPV11 PsV entry. β-Actin was included as loading control. The data are normalized to β-Actin levels and the uninfected control, and expressed as the mean and standard deviation from three biological independent experiments. (**C**, **D**) Western analysis and quantification of p62 and LC3 in NHEKs (**C**) or HaCaT cells (**D**) during HPV11 PsV entry after pre-treatment with the V-ATPase inhibitor bafilomycin A1 (BafA1). The data are normalized to β-Actin levels and the uninfected vehicle control, and expressed as the mean and standard deviation from three biological independent experiments. Significant differences were identified by Student’s t test. *, P < 0.05; **, P < 0.01.

To investigate the activity of the autophagy pathway in cells infected by HPV11 PsVs, the autophagic activity marker proteins LC3 and p62 were examined. Both NHEKs and HaCaT cells were incubated with PsVs for 1 or 2 hours, followed by Western analysis (Figure 3A and 3B). Basic LC3-II/I ratios of uninfected cells indicated that the basal autophagic activity of NHEKs is relatively low compared with HaCaT cells. Furthermore, LC3-II and p62 levels appeared to decline after PsV entry, with reduction in HaCaT cells being more pronounced. Reduction in p62 and LC3-II levels can be explained by two different mechanisms: reduced expression of proteins or autophagy flux resulting in active recycling and degradation in the autolysosome. Therefore, the autophagosome-lysosome fusion step was inhibited by the V-ATPase inhibitor bafilomycin A1 (Baf-A1). Addition of Baf-A1 resulted in accumulation of p62 and LC3-II in both uninfected and infected cells and abolished any differences in p62 and LC3-II levels (Figure 3C and 3D). Therefore, these data indicate that PsV entry in both NHEKs and HaCaT cells stimulates an active autophagy flux and recycling of autophagy marker proteins.

### Autolysosome formation is induced by HPV11 PsV entry

**Figure 4:**
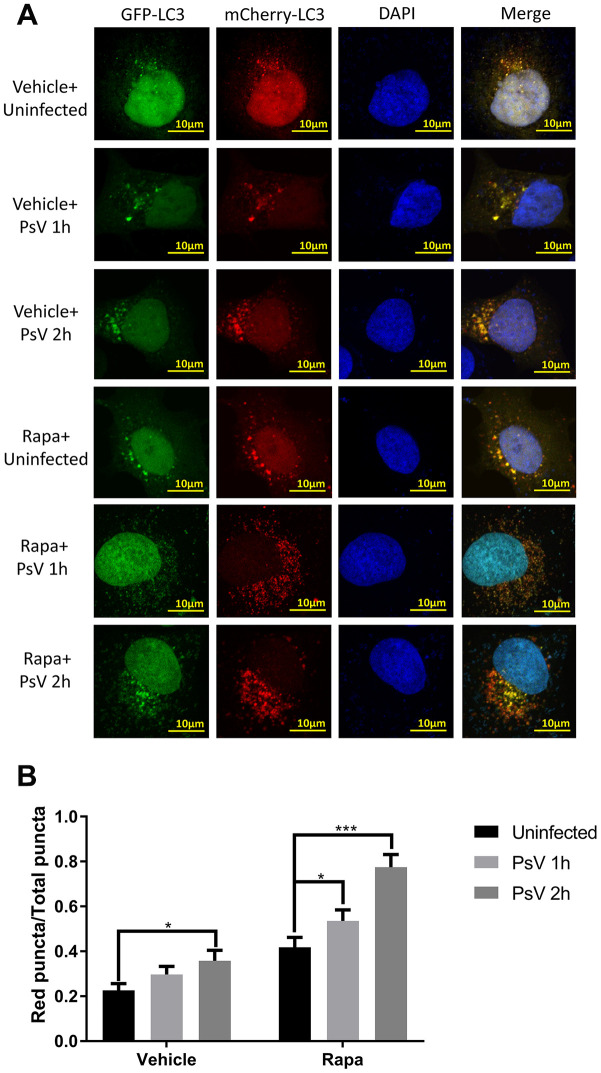
**Autolysosome formation is induced by HPV11 PsVs upon entry of NHEKs and enhanced by rapamycin.** (**A**) Confocal microscopy analysis of HPV11 PsV-infected NHEKs transfected with the mCherry-GFP-LC3 plasmid. Experiments were performed with or without pre-treatment with the canonical autophagy activator rapamycin. (**B**) Quantification of the ratio of red puncta (autolysosomes) to all puncta (autophagosomes plus autolysosomes). The graph represents the mean and standard deviation from three biological independent repeats, and at least 100 cells were analyzed per repeat. Significant differences were identified by Student’s t test. *, P < 0.05; ***, P < 0.005.

As an important process of autophagy, maturation of autophagosomes into autolysosomes was further investigated after transfection with a plasmid expressing the mCherry-GFP-LC3 tandem fusion protein, which is able to distinguish autophagosomes from autolysosomes since the fluorescent activity of GFP is lost in the acidic environment of the autolysosomes while fluorescence of mCherry remains unaffected. Thus, autophagosomes that mature into autolysosomes are visible as red puncta by confocal microscopy. Indeed, increased levels of red puncta were observed upon HPV11 PsV entry in NHEKs compared with uninfected cells (Figure 4A and 4B). Pre-exposure of NHEKs to the canonical autophagy activator rapamycin similarly resulted in increased autophagy flux and autolysosome formation, which was further enhanced after PsV entry (Figure 4A and 4B). These results indicate that induced autophagy flux upon cellular entry by PsVs results in increased autolysosome formation.

### Autophagy reduces HPV11 infections

**Figure 5:**
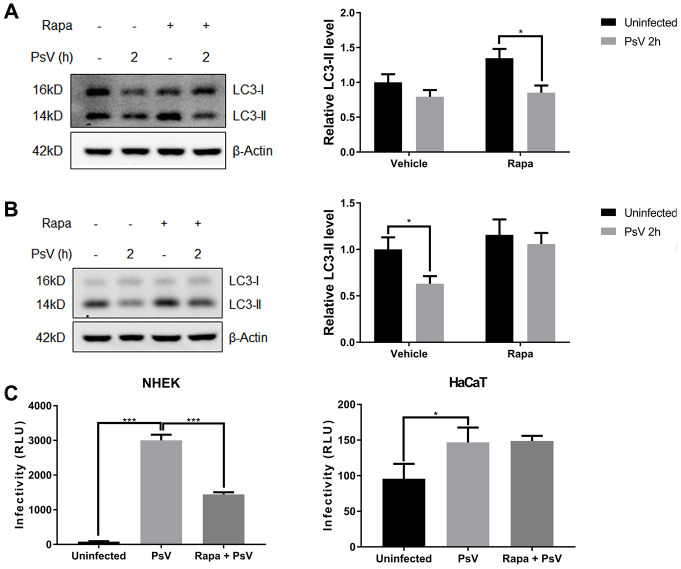
**HPV11 PsV infections are reduced by autophagy.** (**A**, **B**) Western analysis of LC3 in NHEKs (**A**) or HaCaT cells (**B**) during HPV11 PsV entry with or without rapamycin pre-treatment. LC3-II levels are normalized to β-Actin and uninfected vehicle control, and expressed as the mean and standard deviation from three biological independent experiments. (**C**) Infectivity assays using PsVs containing a luciferase expression vector. Luciferase activity is normalized by cell viability and presented as the mean and standard deviation from three biological independent experiments. Significant differences were identified by Student’s t test. *, P < 0.05; ***, P < 0.005.

To evaluate the overall effects of autophagy on HPV11 PsV infectivity, PsVs containing a luciferase-expressing plasmid were used to infect NHEKs and HaCaT cells, which allowed quantification of the infection based on luciferase activity. Furthermore, overall cellular autophagy activity was modulated with the well-established canonical autophagy activator rapamycin. Western analysis showed that in NHEKs, which display low basal autophagy activity, rapamycin pre-treatment significantly enhanced the reduction of LC3-II upon PsV entry (Figure 5A). However in HaCaT cells, which display high basal autophagy activity, no reduction in LC3-II levels were observed upon PsV entry after rapamycin pre-treatment (Figure 5B). These differences are likely related with the basal autophagy activity of the two cell types and the balance between LC3 activation and LC3-II degradation/recycling. Further infectivity assays showed a very strong luciferase signal upon PsV entry of NHEKs, which was significantly reduced in rapamycin pre-treated cells (Figure 5C). In contrast, only a very weak luciferase signal was detected upon PsV entry of HaCaT cells and rapamycin pre-treatment did not appear to have any effect on the luciferase signal. Therefore, these data indicate that autophagy activity is strongly associated with reduced HPV11 infectivity.

### HPV11 PsV entry regulates autophagy through Erk/mTOR and Akt/mTOR signaling

**Figure 6:**
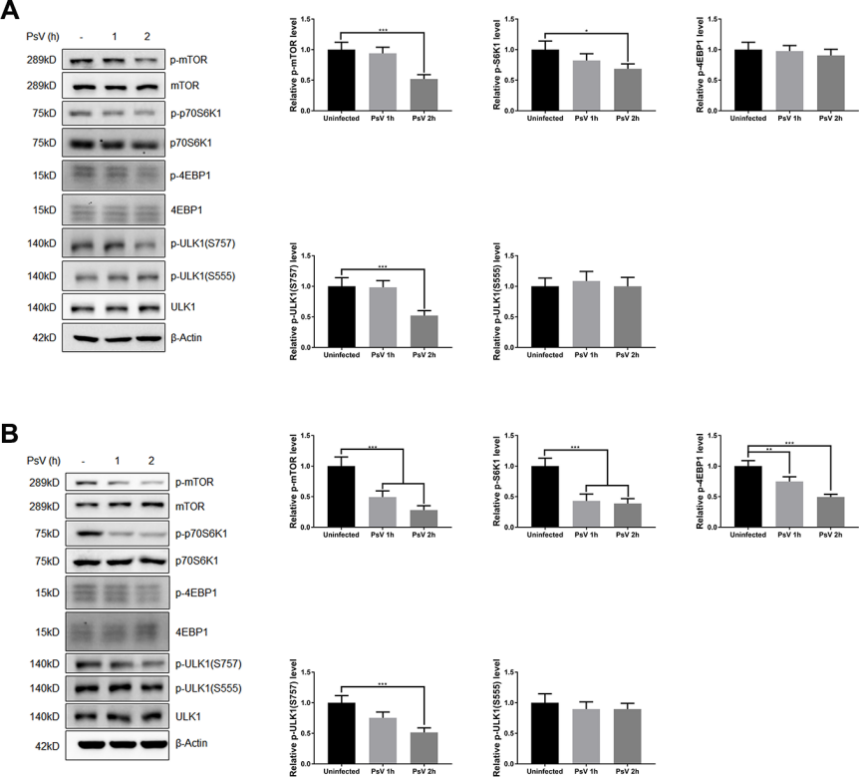
**HPV11 PsV cellular entry activates autophagy through mTOR signaling.** (**A**, **B**) Western analysis of phosphorylated and total mTOR and its substrate proteins 4EBP1, p70S6K1, and ULK1 in NHEKs (**A**) or HaCaT cells (**B**) during HPV11 PsV entry. The ratio of the phosphorylated and total proteins was quantified and normalized to β-Actin and uninfected controls. The graph represents the mean and standard deviation from three biological independent repeats. Significant differences were identified by Student’s t test. *, P < 0.05; **, P < 0.01; ***, P < 0.005.

**Figure 7:**
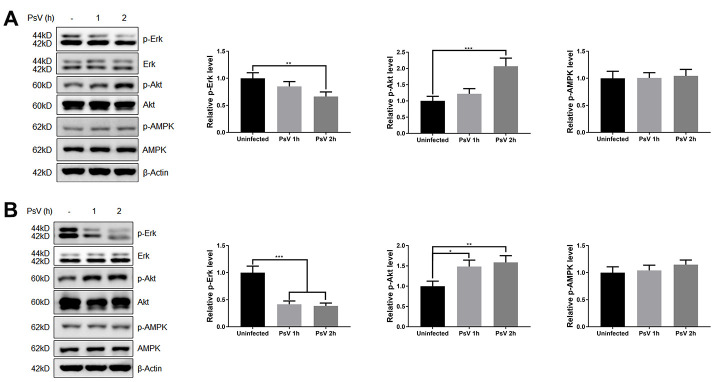
**HPV11 PsV cellular entry regulates Erk and Akt signaling.** (**A**, **B**) Western analysis of phosphorylated and total Erk, Akt, and AMPK in NHEKs (**A**) or HaCaT cells (**B**) during HPV11 PsV entry. The ratio of the phosphorylated and total proteins was quantified and normalized to β-Actin and uninfected controls. The graph represents the mean and standard deviation from three biological independent repeats. Significant differences were identified by Student’s t test. *, P < 0.05; **, P < 0.01; ***, P < 0.005.

**Figure 8:**
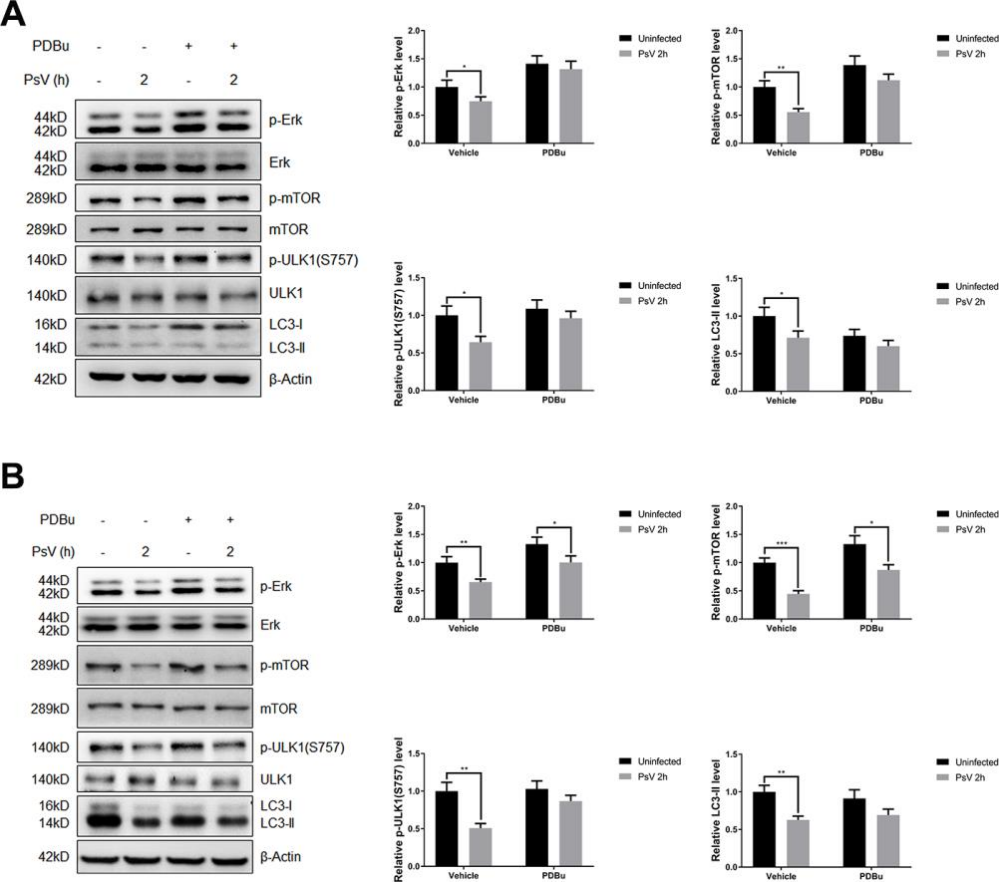
****The inhibition of Erk/mTOR signaling by HPV11 PsVs entry was rescued by PDBu pre-treatment in NHEKs (**A**) or HaCaT cells (**B**). The ratio of the phosphorylated and total proteins was quantified to demonstrate the activity of Erk and mTOR signaling respectively. The graph represents the mean and standard deviation from three independent repeats. Significant differences were identified by Student’s t test. *, P < 0.05; **, P < 0.01; ***, P < 0.005.

Mammalian target of rapamycin (mTOR) is activated by phosphorylation, mainly at Ser2448 as a part of mTOR complex 1 (mTORC1), which inhibits the canonical autophagy pathway by phosphorylation of ULK1 at Ser757. In addition, two non-autophagy-related proteins, 4EBP1 and p70S6K1, are also regulated by mTORC1-dependent phosphorylation. Thus, to elucidate the impact of PsV entry on mTORC1 activity and canonical autophagy the phosphorylation state of mTOR, 4EBP1, p70S6K1, and ULK1 was investigated in NHEKs and HaCaT cells. Western analysis showed that the levels of phosphorylated mTOR, 4EBP1, p70S6K1, and ULK1 (S757) were all reduced after PsVs entry for both cell types (Figure 6), while the levels of phosphorylated ULK1 at position Ser555, which is a target of AMPK, remained unchanged (Figure 6). These results were consistent with elevated autophagy flux induced by HPV11 PsV entry. Moreover, inhibition of mTOR signaling in HaCaT cells was more pronounced compared with NHEKs, which is also consistent with the observed LC3-II and p62 levels. To further elucidate the upstream pathways involved in inhibition of mTOR signaling upon PsV entry, phosphorylation levels were investigated for MAPK/Erk, Akt and AMPK. Cellular entry by PsVs resulted in reduced phosphorylation levels of MAPK/Erk and increased phosphorylation of Akt, while no differences in phosphorylated AMPK were observed (Figure 7). As we expected, the chemical activator of Erk (phorbol 12,13-dibutyrate, PDBu) pre-treatment restored the phosphorylation levels of Erk and partially rescued the phosphorylation levels of mTOR and ULK1 (S757). In addition, the autophagy activity marker protein LC3-II was partially restored (Figure 8). Therefore, these results show that induced autophagic activity upon cellular entry of PsVs is regulated by a balance in MAPK/Erk and Akt signaling.

## DISCUSSION

Viruses have developed different strategies to modulate the host autophagic response in order to create a more conducive environment for viral replication [15]. Several conflicting results have been reported regarding the regulation of autophagy during cellular entry by HPV16 virions. In HaCaT cells, activation of phosphatidylinositol 3-kinase (PI3K)/Akt/mTOR signaling inhibited autophagy in the early stages of HPV16 infection [20]. Whereas in primary human foreskin keratinocytes (HFKs), HPV16 virions significantly induced autophagy [19]. Furthermore, it has also been demonstrated that autophagy was induced in HeLa cells during the HPV16 entry process [21]. Therefore, it appears that autophagic responses against HPV16 are cell-type dependent, similar to what was also previously shown for HPV16 internalization and trafficking in host cells. Some studies showed that HPV16 entry requires clathrin-dependent endocytosis [22–25], however, other studies showed that HPV16 internalization requires tetraspanin-enriched microdomains for endocytosis, which was independent of clathrin [26, 27]. Our previous study has revealed that HPV11 E6 induces autophagy by suppressing the AKT/mTOR and Erk/mTOR pathways, suggesting elevated autophagy level is beneficial for the survival of HPV11 in host cells after successful infection [28]. However, the host autophagic response during HPV11 entry has not been reported thus far. In our study, both primary and immortalized keratinocytes (NHEK and HaCaT) were used to investigate host autophagic responses to HPV11 entry.

Previous analysis of intracellular HPV16 PsVs in HeLa cells by TEM showed the presence of double-membrane vesicles, which are typical structures of autophagosomes, containing 10-50 PsV particles in the lumen [21]. In our study, TEM analysis of NHEKs incubated with HPV11 PsVs also showed the presence of double-membrane vesicles containing PsV particles, although the number of particles per vesicle appeared to be lower than reported for the HPV16 PsVs [21]. Previous studies showed stronger autophagy induction by HPV16 in cells with higher basal autophagy levels, which was inversely correlated with HPV16 infectivity [19], and pre-treatment with an mTOR inhibitor significantly reduced HPV16 infections [20]. In our study, HPV11 PsVs induced stronger autophagic responses in HaCaT cells, which displayed higher basal autophagy levels, and resulted in lower infectivity of HPV11. Furthermore, induction of basal autophagy levels in NHEKs by rapamycin enhanced the autophagy response to HPV11 PsVs and reduced HPV11 infectivity, which further confirmed the correlation between basal autophagy levels and HPV infectivity.

Analysis of autophagy regulatory cues demonstrated mTOR signaling and ERK phosphorylation were reduced, while Akt phosphorylation was induced after HPV11 entry, suggesting autophagy was induced through mTOR inhibition, likely via Erk regulation. However, Akt activation might have contributed to the overall autophagy levels with opposing signals, indicating that the autophagy levels were the results of a balance in both activating and repressing signals. The Ras/Erk and PI3K/Akt/mTOR signaling pathways are chief mechanisms for controlling cell survival, differentiation, proliferation, metabolism, and motility in response to extracellular cues [29]. The Ras/Erk and PI3K/Akt pathways have been shown to negatively regulate each other’s activity. For instance, a cross-inhibition signal between Akt and Erk is induced by strong insulin growth factor-1 (IGF1) stimulation [30]. Akt negatively regulates Erk activation by phosphorylating inhibitory sites in the Raf N-terminus [31–34]. It has previously been demonstrated that HPV16 exposure activates PI3K/Akt signaling from growth factor receptors (GFRs) in HaCaT cells [20, 35], therefore, Erk may actually be downregulated by Akt activation.

In conclusion, we report here that HPV11 PsV interaction with human keratinocytes induces autophagy through reduction of Erk/mTOR signaling. As natural host cells of HPVs, primary human keratinocytes (NHEKs) were more vulnerable to HPV11 infection due to weaker autophagic responses, which is correlated with lower basal autophagy level. Recently, a clinical trial investigating topical rapamycin demonstrated reduced markers of senescence and aging on human skin [36]. Our findings now also suggest that rapamycin pre-treatment may be a potential therapy to prevent the recurrence of condyloma acuminatum by helping to eliminate latent HPV11 infection in keratinocytes.

## MATERIALS AND METHODS

### Cell culture and reagents

Normal human epidermal keratinocytes (NHEKs, passage 4; ScienCell Research Laboratories) were cultured in EpiLife medium (Gibco) containing 10% fetal bovine serum (Gibco) and 293FT and HaCaT cells (Jennio Biotech) were cultured in Dulbecco’s modified Eagle’s medium (DMEM) (Life Technologies) containing 10% fetal bovine serum (Gibco) at 37°C in 5% CO_2_. When appropriate, cells were seeded in a 6-well plate at the density of 1×10^6^/well, and then pre-treated with 25 nM bafilomycin A1 (Baf-A1; MCE, Cat#: HY-100558) for 6h, with 100 nM rapamycin (Sigma, Cat#: V900930-1MG) for 12h or with 1 μM phorbol 12,13-dibutyrate (PDBu; MCE, Cat#: HY-18985) for 30 min before PsV infection.

### Production of HPV11 PsVs

The production of HPV11 PsVs was based on HPV16 PsV production methods described previously [37–39]. Briefly, 293FT cells were co-transfected with two plasmids expressing HPV11 capsid proteins L1 and L2 and a luciferase reporter plasmid following the ratio p11L1w:p11L2w:pGM-CMV-Lu=10:1:15. Plasmid constructs p11L1w and p11L2w were kindly provided by Dr. Martin Müller of the German Cancer Research Center (DKFZ).

### Transmission electron microscopy

NHEKs were incubated with 1,000 vge/cell of HPV11 PsVs and fixed with 2.5% glutaraldehyde. Cells were subsequently incubated with 2% osmium tetroxide in 0.1 M sodium cacodylate buffer and embedded in epoxy resin. Sections were cut at a nominal thickness of 80 nm and stained with uranyl acetate and lead citrate. Sections were examined at 120 kV using a Tecnai G2 Spirit transmission electron microscope (FEI Company).

### Immunofluorescence and confocal microscopy

NHEKs were incubated with 1,000 vge/cell of HPV11 PsVs for indicated times, washed with phosphate-buffered saline (PBS), and fixed with 4% paraformaldehyde (Solarbio) for 15 min. The fixed cells were permeabilized with 0.1% Triton X-100 (Solarbio) for10 min followed by blocking with 10% goat serum in 1% bovine serum albumin for 30 min at room temperature and incubating with polyclonal rabbit anti-HPV11 L1 IgG (1:200 dilution; Biodragon) overnight at 4°C. Cells were subsequently washed and incubated with Alexa Fluor 594 donkey anti-rabbit IgG (1:400 dilution; Yeasen) for 30 min in the dark. Finally, the specimens were retained in mounting medium with DAPI (Abcam) for 5 min at room temperature in the dark, and images were obtained using a Nikon A1 confocal microscope.

### Western blot analysis

Cells were incubated with 1,000 vge/cell of HPV11 PsVs for indicated times and approximately 30 μg of cell lysate protein were subjected to western blotting protocols as described previously [28], using the following antibodies: polyclonal rabbit anti-LC3B (Cat#: L7543) and monoclonal mouse anti-actin (Sigma, Cat#: A9357), monoclonal rabbit anti-p62 (MBL Life science, Cat#: PM045), monoclonal rabbit anti-phospho-mTOR (Ser2448, Cat#: 2971), monoclonal rabbit anti-mTOR (Cat#: 2983), anti-phospho-70S6 kinase (Cat#: 9234), monoclonal rabbit anti-70S6 kinase (Cat#: 2708), monoclonal rabbit anti-phospho-4E-BP1 (Thr37/46, Cat#: 2855), monoclonal rabbit anti-4E-BP1 (Cat#: 9452), monoclonal rabbit anti-Erk (Cat#: 4695), monoclonal rabbit anti-phospho-Erk (Thr202/Tyr204, Cat#: 4370), monoclonal rabbit anti-Akt (Cat#: 4691), monoclonal rabbit anti-phospho-Akt (Ser473, Cat#: 4060), monoclonal rabbit anti-ULK1 (Cat#: 8054), monoclonal rabbit anti-phospho-ULK1 (Ser555, Cat#: 5869), monoclonal rabbit anti-phospho-ULK1 (Ser757, Cat#: 14202), monoclonal rabbit anti-AMPK (Cat#: 2603) and monoclonal rabbit anti-phospho-AMPK (Ser172, Cat#: 2531) (Cell Signaling Technology).

### Infectivity assays

To measure HPV infectivity, cells were incubated with 1,000 vge/cell HPV11 PsVs containing a luciferase-expressing plasmid and luciferase activity was measured at 24h post infection, using the Luciferase Assay System (Promega) according to the manufacturer’s instructions. In a parallel experiment, relative cell viability was measured using the Cell Counting Kit-8 (Dojindo) according to the manufacturer’s instructions.

### Statistical analysis

All data are expressed as mean ± standard deviation derived from three independent replicate experiments. Statistical differences were determined using Student’s t test. Data groups are considered statistically significant if P values < 0.05.
